# Supplementary material for: Nitrogen Fertilization Shapes Soil Microbial Diversity and Ecosystem Multifunctionality by Modulating Soil Nutrients
Source: Microorganisms. 2025 Feb 27;13(3):540. doi: 10.3390/microorganisms13030540 (PMC11944318; doi:10.3390/microorganisms13030540)
Supplement: Supplementary file 1 [file microorganisms-13-00540-s001.zip › microorganisms-3488218-supplementary.pdf]

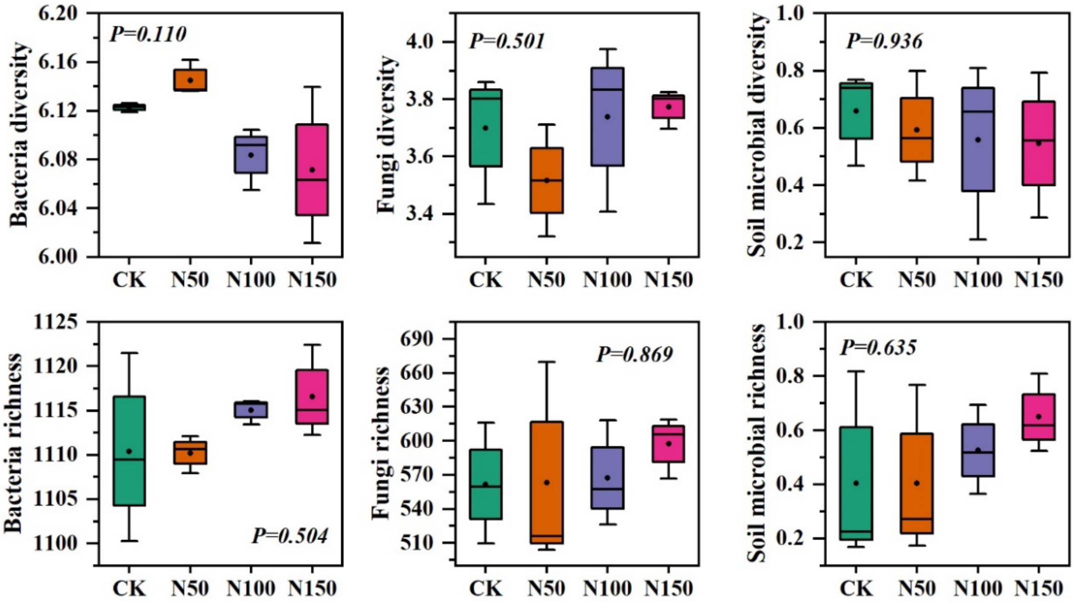

S1. Alpha diversity index of soil microbial community under different nitrogen fertilizer treatments
